# A comparison and evaluation of five biclustering algorithms by quantifying goodness of biclusters for gene expression data

**DOI:** 10.1186/1756-0381-5-8

**Published:** 2012-07-23

**Authors:** Li Li, Yang Guo, Wenwu Wu, Youyi Shi, Jian Cheng, Shiheng Tao

**Affiliations:** 1 State Key Laboratory of Crop Stress Biology in Arid Areas and College of Sciences, Northwest A&F University, Yangling, Shaanxi, 712100, China; 2 College of Life Sciences, Northwest A&F University, Yangling, Shaanxi, China; 3 Institute of Applied Mathematics, Northwest A&F University, Yangling, Shaanxi, China; 4 Bioinformatics Center, Northwest A&F University, Yangling, Shaanxi, China

## Abstract

**Background:**

Several biclustering algorithms have been proposed to identify biclusters, in which genes share similar expression patterns across a number of conditions. However, different algorithms would yield different biclusters and further lead to distinct conclusions. Therefore, some testing and comparisons between these algorithms are strongly required.

**Methods:**

In this study, five biclustering algorithms (i.e. BIMAX, FABIA, ISA, QUBIC and SAMBA) were compared with each other in the cases where they were used to handle two expression datasets (GDS1620 and pathway) with different dimensions in *Arabidopsis thaliana* (*A. thaliana*)

GO (gene ontology) annotation and PPI (protein-protein interaction) network were used to verify the corresponding biological significance of biclusters from the five algorithms. To compare the algorithms’ performance and evaluate quality of identified biclusters, two scoring methods, namely weighted enrichment (WE) scoring and PPI scoring, were proposed in our study. For each dataset, after combining the scores of all biclusters into one unified ranking, we could evaluate the performance and behavior of the five biclustering algorithms in a better way.

**Results:**

Both WE and PPI scoring methods has been proved effective to validate biological significance of the biclusters, and a significantly positive correlation between the two sets of scores has been tested to demonstrate the consistence of these two methods.

A comparative study of the above five algorithms has revealed that: (1) ISA is the most effective one among the five algorithms on the dataset of GDS1620 and BIMAX outperforms the other algorithms on the dataset of pathway. (2) Both ISA and BIMAX are data-dependent. The former one does not work well on the datasets with few genes, while the latter one holds well for the datasets with more conditions. (3) FABIA and QUBIC perform poorly in this study and they may be suitable to large datasets with more genes and more conditions. (4) SAMBA is also data-independent as it performs well on two given datasets. The comparison results provide useful information for researchers to choose a suitable algorithm for each given dataset.

## Background

In recent years, with the development of high throughput technologies such as the gene microarray and next-generation sequencing, advanced analysis tools are required to extract information from the huge amount of data. Clustering genes according to their expression profiles is an important technique in extracting knowledge from microarray data. Usually, gene expression data is arranged in a data matrix, where rows represent genes and columns represent conditions.

Traditional clustering techniques like hierarchical clustering [1] and k-means clustering work well for small data sets but perform poorly when the number of experimental conditions is large since these methods cluster the genes based on their expression under all conditions. In fact, many activation patterns are common to a group of genes only under specific experimental conditions. Besides, clusters generated by these algorithms can not overlap, i.e. a gene belongs to at most one cluster, whereas in fact the gene may participate in different activation patterns for different conditions. To move beyond these limits, a modified clustering concept called biclustering has been suggested in several studies [2-8].

A survey of biclustering algorithms has been given by Madeira and Oliveira [9]. The biclusters are defined to be a set of genes and a set of conditions, in which these genes may involve in similar biological processes under these specific conditions. Moreover, biclusters can overlap on both genes as well as conditions.

Several biclustering algorithms for microarray expression data have been proposed recently [7,10,11]. However, there is few comparison among different algorithms, making it hard for researchers to make a rational choice among them. Ayadi et al. [12] compared biclustering algorithms mainly by using idealized simulated data, which may not be the case in the real data sets since real expression data sets are larger and more complex. Therefore, we have chosen two real expression datasets (GDS1620 and pathway) in our study, which are both selected from *A. thaliana*. The comparison results based on them would be more comparable.

We have chosen five well established biclustering algorithms for our comparative study according to three criteria: (1) to what extent the algorithm has been used or referenced in this field; (2) whether an implementation is available; (3) whether the algorithm is considered to be novel. The selected algorithms are BIMAX [5], FABIA(Factor Analysis for Bicluster Acquisition) [13], ISA (Iterate Signature Algorithm) [3], QUBIC (Qualitative Biclustering algorithm) [14] and SAMBA (Statistical-Algorithmic Method for Bicluster Analysis) [4].

For real transcriptome data sets, the most meaningful verification of biclusters is biological interpretation. Prelic et al.’s [5] verification was based on the number of gene ontology(GO) terms enriched for the biclusters. Li et al. [14] recorded the best *p-value* of the GO term as the significant level value of the bicluster. These two methods are obviously inappropriate, as the number of GO terms and the significance levels of enriched GO terms are dependent on bicluster size. Besides, genes that have not been annotated may affect the results in these situations. Therefore, in order to compare the biclustering results of different algorithms objectively and quantitatively, we proposed a new weighted enrichment (WE) scoring method and protein-protein interaction network scoring method [15]. For each dataset, by applying one of our scoring methods (WE and PPI) to biclusters generated by the five algorithms, we got a set of scores. Then, we combined all biclusters into a single ranking according to the overall scores. Finally, we used the distribution of the biclusters by each algorithm in the different sections of the ranking as the criterion to evaluate the algorithm, which would be very helpful in analyzing the difference of the algorithms.

## Methods

### Datasets

Two datasets were used to test these five algorithms, GDS1620 and metabolic pathway dataset for *A. thaliana*. The former was downloaded from GEO [16], and the latter was downloaded from [17]. Since the two gene expression datasets are for *A. thaliana*, the results based on them would be comparable.

The dataset of GDS1620 is about abiotic stress-inducing agents effect on suspension cell cultures. It contains expression profiles of 22810 probe sets under 37 conditions. The Bioconductor [18] and R [19] software were used to pre-process the dataset GDS1620 including nonspecific filtering; removing the control probe sets and duplicated probe sets. After the pre-processing, there were only 3881 probe sets and 16 conditions left.

The dataset of metabolic pathway contains expression profiles of 734 genes under 69 conditions.

### Selected algorithms

Five biclustering algorithms (i.e. BIMAX, FABIA, ISA, QUBIC and SAMBA) were chosen for comparison, the implementations of which were all available from the original publications. Among these algorithms, BIMAX, ISA and SAMBA have been used or referenced frequently in previous studies. In contrast, FABIA and QUBIC are relatively new methods and the comparisons are more valuable.

### Gene ontology weighted enrichment score

For real transcriptome datasets, the most meaningful evaluation of biclusters is biological interpretation.

For each identified bicluster, we used the cytoscape plugin, i.e. BiNGO [20], to perform GO enrichment analysis in biological processes namespace. Hyper geometric tests were used for statistical analysis and the Benjamin-Hochberg False Discovery Rate (FDR) procedure [21] was used for the multiple tesing corrections. We selected 0.05 as significance level.

*P-value* is the probability of that *x* number of genes from a bicluster of size *X* annotated to a particular GO term, given *P* which is the proportion of genes in the whole genome annotated to that GO term. So the *p-value* can be evaluated using the following hyper-geometric function [22],

(1) $$p-value=1-\sum_{i=1}^{x-1}\frac{\left(\begin{array}{l} PN\\ i \end{array}\right)\left(\begin{array}{l} N-PN\\ X-i \end{array}\right)}{\left(\begin{array}{l} N\\ X \end{array}\right)}$$

where *N* is the total number of genes in the whole genome. The closer the *p-value* is to zero; the more significant is the association of the particular GO term with the group of genes.

For all GO terms significantly associated with a bicluster, we processed the *p-value* of every GO term on –log scale as the enrichment score of this GO term, and then used the weighted mean of these scores as enrichment score of this bicluster. As a matter of fact, the GO term associated with more genes may not have higher enrichment score, instead, it accounts for more proportions of genes in the bicluster. So we consider this term contribute more to the enrichment score of this bicluster and the weight of each GO terms is$x_{i}/X$, where $x_{i}$ is the number of genes in this bicluster significantly annotated to the *i-th* GO term and *X* is the total number of genes belonging to the bicluster which contains three parts: (1) genes enriched to a GO term; (2) genes that have not been annotated; and (3) genes that are not enriched to any GO term but have been annotated. Therefore, the WE score of this bicluster is described as:

(2) $$\begin{array}{l} WE-score=\frac{s_{1}x_{1}/X+s_{2}x_{2}/X+\cdots +s_{n}x_{n}/X+non*0/X}{x_{1}/X+x_{2}/X+\cdots +x_{n}/X+non/X}\\ =\frac{x_{1}s_{1}+x_{2}s_{2}+\cdots +x_{n}s_{n}}{x_{1}+x_{2}+\cdots +x_{n}+non} \end{array}$$

(3) $$s_{i}=-\log(p_{i})$$

where $p_{i}$ is the *p-value* of the *i-th* GO term; *n* is the number of GO terms to which the genes from this bicluster are significantly enriched; *non* is the number of genes which are not significantly enriched to any GO term but have the annotation. From the expression of the WE score, we can see that the value of WE score do not have relationship with *X*, i.e. WE score does not have relationship with no annotation genes. So, the higher WE score is; the more biologically significant the bicluster would be.

### Protein-protein interaction score

Interactions between proteins provide a basis for most biological processes in an organism [23], and hence the networks formed by interacting proteins provided us with crucial platform to analysis the physical and functional association in various biological processes. In this study, we used the protein-protein interaction networks to assess the quality of the biclusters, as genes that show similar expression patterns may participate in the same interaction network. In order to compare the biclusters from different algorithms, we proposed a PPI (protein-protein interaction) scoring method.

In this work, we localized the PPI of *Arabidopsis thaliana* from database STRING (http://string-db.org/) [24], which integrates and weights information from numerous sources, including conserved neighborhood, gene fusions, phylogenetic co-occurrence, co-expression, database imports(e.g. MINT, HPRD, BIND, DIP, BioGRID, KEGG and Reactome), large-scale experiments, literature co-occurrence [25]. Interactions from these data sources are benchmarked and scored against a common reference that joints membership of proteins in biological pathways, as annotated at KEGG [26]. The scores higher than 0.7 will be considered as high confidence, and the confidence increases when methods were combined [25]. We took the interactions between two genes with combined scores higher than 0.7 into consideration.

The PPI score of a bicluster is calculated by the following expression,

(4) $$PPI-score=\frac{I}{N-M}$$

where *I* is the number of genes which have interaction relationship with other genes in the same bicluster, *N* is the total number of genes in this bicluster, and *M* is the number of genes in this bicluster which have not been found to interact with any genes according to all data in STRING database.

## Results

We implemented the five algorithms on two real datasets described above part respectively. BIMAX, ISA and FABIA were applied respectively using three R packages: biclust [27,28], isa2 [29] and fabia [13]; meanwhile, QUBIC used qubic0.21 package, and SAMBA was performed by Expander package [30]. The parameter settings of these algorithms, which were summarized in Table1, were set optimally according to previous studies and our tests.

**Figure 1:**
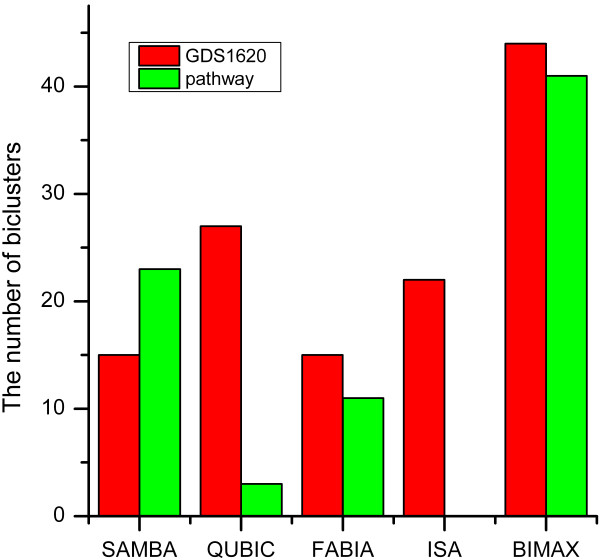
**Number of biclusters.** The number of biclusters generated by different algorithms for two different datasets after filtering out small biclusters was shown for comparison.

The biclusters with fewer than 2 conditions or 5 genes were filtered out from bicluster lists obtained from GDS1620, and we also filtered out the biclusters with fewer than 3 conditions or 5 genes obtained from pathway data. After filtering, the number of biclusters for each dataset was shown in Figure1 and the details were summarized in the Additional file 1: Table S1.

**Table 1:** Compared biclustering algorithms and their parameter settings

**Method**	**GDS1620 datasets**	**Pathway datasets**
***BIMAX***	minr = 5, minc = 2	Minr = 5, minc = 3
***FABIA***	p = 16, alpha = 0.1, cyc = 500	p = 50, alpha = 0.1, cyc = 500
***ISA***	no.seeds = 13	no.seeds = 50
***QUBIC***	k = 5, f = 0.1, c = 0.95, o = 50, q = 0.06, r = 2	k = 5, f = 0.5, c = 0.65, o = 25, q = 0.1, r = 2
***SAMBA***	opt = valsp_3ap, overlap = 0.1, max = 4	opt = valsp_3ap, overlap = 0.1, max = 7

The five algorithms’ parameters were set optimally according to previous studies and our tests for different datasets.

We compared performance of these algorithms based on three criteria: 1) the number of biclusters generated by an algorithm; 2) ranking of the biclusters generated by an algorithm in the combined ranking of all biclusters generated by all algorithms based on WE scores; 3)ranking of the biclusters generated by an algorithm in the combined ranking of all biclusters generated by all algorithms based on PPI scores.

### Comparison based on the number of biclusters

From the Figure1, we could find that SAMBA output the similar number of biclusters on two different data sets, and so did FABIA, but both QUBIC and ISA had very different performances on these two different data sets. In particular, ISA returned 22 biclusters for GDS1620 dataset, but no bicluster for dataset of pathway. The performance of QUBIC might also depend on the size of the dataset it used. BIMAX could not be evaluated by this criterion as the number of biclusters was a predefined parameter to the implementation of the algorithm.

### Functional enrichment

**Figure 2:**
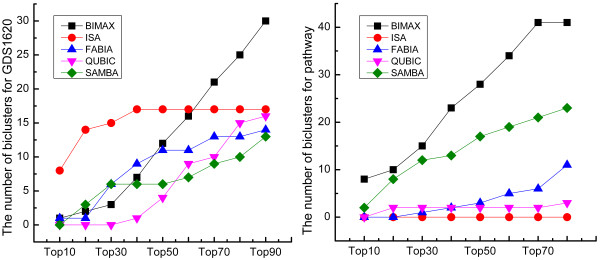
**Rank distributions of biclusters based on GO WE scores.** Rank distributions of biclusters from each algorithm in a combined ranking based on Gene Ontology WE scores for two different datasets.

In order to evaluate the quality of the biclusters quantitatively, we computed the WE scores of every bicluster using GO WE scoring method. For each dataset, we combined all biclusters into a single ranking based on their WE scores. Then, we obtained the distribution of the biclusters output by each algorithm in this unified ranking as shown in the Figure2. The details about WE scores of all biclusters for two datasets were summarized in Additional file 2: Table S2 and Additional file 3: Table S3.

For dataset GDS1620, ISA achieved the highest scores than any other algorithms. BIMAX, FABIA and SAMBA achieved middle scores just inferior to ISA. For dataset of pathway, BIMAX tended to achieve the highest WE scores than any other algorithms, and the second algorithm with relatively high scores was SAMBA. In contrast, the scores for QUBIC were consistently low on two datasets due to the same reason as discussed in the previous section that this algorithm might be size-dependent on dataset.

### Protein-protein interaction network

**Figure 3:**
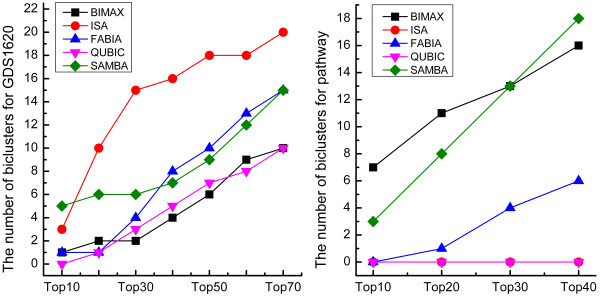
**Rank distributions of biclusters based on PPI scores.** Rank distributions of biclusters from each algorithm in a combined ranking based on PPI scores for two different datasets.

We also used PPI scoring to evaluate the quality of the biclusters quantitatively. For each dataset, we first calculated the PPI score of each bicluster and combined all biclusters into a single ranking based on their PPI scores. Then, we obtained the distribution of the biclusters generated by all algorithms in this unified ranking as shown in the Figure3. The details about the PPI scores of the biclusters for two datasets were described in Additional file 2: Table S2 and Additional file 3: Table S3.

For GDS1620 dataset, the biclusters output by ISA appeared to have the highest PPI scores compared to other algorithms, once again endorsing the fact that the biclusters of ISA were more biologically significant than those of other algorithms. The scores of biclusters generated by SAMBA was moderately high just inferior to those of ISA. For other three algorithms (i.e. BIMAX, FABIA and QUBIC), the biclusters had low scores with a slight advantage of FABIA over BIMAX and QUBIC. For dataset of pathway, biclusters of BIMAX algorithm tended to have the highest PPI scores than those of any other algorithms. And the scores of the biclusters generated by SAMBA were comparable to those of BIMAX. By contrast, both FABIA and QUBIC performed poorly, and might be suitable for much larger datasets.

### Comparison based on random gene groups

**Figure 4:**
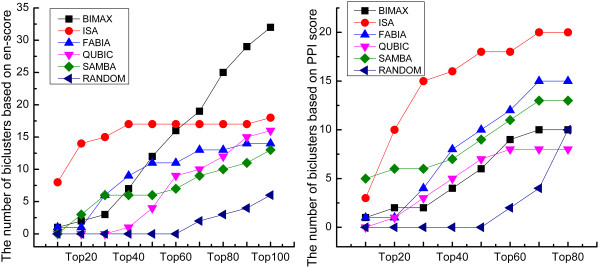
**Rank distributions of biclusters and random gene groups.** Rank distributions of biclusters from each algorithm and random gene groups in a combined ranking based on two scores (i.e. WE scores and PPI scores) for dataset of GDS1620.

To validate the efficiency of all algorisms against random gene groups, a simulation study was performed to randomly draw 15 subsets of GDS1620 with different genes and conditions. We calculated WE scores and PPI scores of these 15 random gene groups (Additional file 4: Table S4), and combined these scores with those of the biclusters generated by the five algorisms. The rank distributions of the biclusters and random gene groups were shown in the Figure4. The biclusters generated by the five algorithms were significantly different to random gene groups, and had higher WE scores and PPI scores than random gene groups. This indicated that these algorisms were very effective to find biologically significant gene groups.

### Correlation analysis between WE scores and PPI scores

Although Gene Ontology annotations and protein-protein interaction networks are derived from different types of data, one can expect that WE scores and PPI scores of the biclusters are statistically consistent. To validate this consistency, we applied *Kendall tau rank correlation coefficient*[31] to test the association between the paired scores. In the result, the *tau* was *0.4318* and *p-value* was *4.714e-11*, which indicates that the two scores are positively associated.

## Discussion and conclusions

In this study, we compared five well-established biclustering algorithms to evaluate their capabilities of identifying biologically significant groups of co-expressed genes under a number of conditions. The evaluation criteria of biological significance for biclusters used in our study were GO annotation and protein-protein interaction network. In order to compare the performance of the algorithms objectively and quantitatively, we proposed two methods: GO WE scoring and PPI scoring. The biclusters of all algorithms has better performances than the random gene groups.

From the ranking of the biclusters based on the WE scores and PPI scores (Figures. 2 and 3), we find that the distributions of biclusters for each algorithm based on these two sets of scores are almost consistent. Moreover, *Kendall tau rank correlation coefficient* test shows that there is significantly positive association between two lists of scores. Hence, it can be confirmed that the two scoring methods are both effective up to a certain degree.

In our study, the results are generally consistent with several other surveys of biclustering algorithms. Like Prelic et al. [5] and Richards et al. [32], we find that ISA is an effective algorithm that can generate biclusters with high GO WE scores and PPI scores for large dataset (GDS1620). For dataset of pathway, like result from Chia et al. [33], ISA algorithm returned no bicluster, which was attributed to the fact that this dataset contains too few conditions. However, their conclusion is not consistent with our results, because 22 biclusters have been identified on dataset GDS1620 which has fewer conditions. It suggests that ISA is gene size-dependent, and it is not suitable for the dataset with few genes. In this study, we also find that SAMBA performed well which is consistent with the results of [5] and [33], and it might be less data-dependent. For BIMAX, the biclusters has high scores only for dataset of pathway, which indicates that this algorithm holds for the dataset with more conditions. FABIA and QUBIC perform poorly in the study, and this may be attributable to the fact that the datasets used here were much smaller in size. Thus, such two algorithms might be suitable for a large dataset with more genes and more conditions.

Our results will provide researchers with useful information to make a rational choice among the algorithms according to datasets to be used. In addition, the two scoring methods are useful to provide quantitative and objective assessment for the goodness of biclusters and performance of biclustering algorithms in identifying biologically significant biclusters.

## Competing interests

The authors declare that they have no competing interests.

## Authors' contributions

LL, ST and YG conceived and designed the program. LL and WW wrote the paper. YG, JC and SY constructively evaluated and edited the paper. All authors read and approved the final manuscript.

## Supplementary Material

Additional file 1 **Additional file 1: Table S1.** The number of biclusters output by the five algorithms. This table showed the implementations of the compared five biclustering algorithms and the number of biclusters they output for datasets of GDS1620 and pathway. The biclusters with fewer than 2 conditions or 5 probes were filtered out from all biclusters for dataset of GDS1620. And we also filtered out the biclusters with fewer than 3 conditions or 5 probes for dataset of pathway. Click here for file

Additional file 2 **Additional file 2: Table S2.** WE-scores and PPI scores of all biclusters obtained from dataset of GDS1620. This table showed the WE scores and PPI scores of all biclusters output by the five biclustering algorithms upon GDS1620 dataset. In the table, the biclusters’ names prefixed with ‘b’ referred to the biclusters output by BIMAX algorithm, ‘f’ referred to FABIA algorithm, ‘is’ referred to ISA algorithm, ‘q’ referred to QUBIC algorithm, and ‘s’ referred to SAMBA algorithm. Click here for file

Additional file 3 **Additional file 3: Table S3.** WE-scores and PPI scores of all biclusters generated from dataset of pathway. This table showed the WE scores and PPI scores of all biclusters output by the five biclustering algorithms upon dataset of pathway. In the table, the biclusters’ names prefixed with ‘b’ referred to the biclusters output by BIMAX algorithm, ‘f’ referred to FABIA algorithm, ‘is’ referred to ISA algorithm, ‘q’ referred to QUBIC algorithm, and ‘s’ referred to SAMBA algorithm. Click here for file

Additional file 4 **Additional file 4: Table S4.** WE-scores and PPI scores of random gene groups generated from dataset of GDS1620. This table showed the WE scores and PPI scores of 15 random gene groups generated from GDS1620 dataset. Click here for file
