# Supplementary material for: A comparison and evaluation of five biclustering algorithms by quantifying goodness of biclusters for gene expression data
Source: BioData Min. 2012 Jul 23;5:8. doi: 10.1186/1756-0381-5-8 (PMC3447720; doi:10.1186/1756-0381-5-8)
Supplement: Additional file 1 — Table S1. The number of biclusters output by the five algorithms. This table showed the implementations of the compared five biclustering algorithms and the number of biclusters they output for datasets of GDS1620 and pathway. The biclusters with fewer than 2 conditions or 5 probes were filtered out from all biclusters for dataset of GDS1620. And we also filtered out the biclusters with fewer than 3 conditions or 5 probes for dataset of pathway. [file 1756-0381-5-8-S1.docx]

### Additional table 1 – The number of biclusters output by the five algorithms.

This table showed the implementations of the compared five biclustering algorithms and the number of biclusters they output for datasets of GDS1620 and pathway. The biclusters with fewer than 2 conditions or 5 probes were filtered out from all biclusters for dataset of GDS1620. And we also filtered out the biclusters with fewer than 3 conditions or 5 probes for dataset of pathway.

| **Method** | **Implementation** | **GDS1620 datasets** | **Pathway datasets** |
| --- | --- | --- | --- |
| ***BIMAX*** | R language | 44 | 41 |
| ***FABIA*** | Bioconducter | 15 | 11 |
| ***ISA*** | R language | 22 | 0 |
| ***QUBIC*** | C language | 27 | 3 |
| ***SAMBA*** | Expander | 15 | 23 |
